# FMAj: a tool for high content analysis of muscle dynamics in *Drosophila *metamorphosis

**DOI:** 10.1186/1471-2105-15-S16-S6

**Published:** 2014-12-08

**Authors:** Yadav Kuleesha, Wee Choo Puah, Feng Lin, Martin Wasser

**Affiliations:** 1 School of Computer Science, Nanyang Technological University, N4-2A-05, Nanyang Avenue, Singapore 639798; 2 Imaging Informatics Division, Bioinformatics Institute (BII), Agency for Science, Technology and Research (A*STAR), 30 Biopolis Street, #07-01 Matrix, Singapore 138671

## Abstract

**Background:**

During metamorphosis in *Drosophila melanogaster*, larval muscles undergo two different developmental fates; one population is removed by cell death, while the other persistent subset undergoes morphological remodeling and survives to adulthood. Thanks to the ability to perform live imaging of muscle development in transparent pupae and the power of genetics, metamorphosis in *Drosophila *can be used as a model to study the regulation of skeletal muscle mass. However, time-lapse microscopy generates sizeable image data that require new tools for high throughput image analysis.

**Results:**

We performed targeted gene perturbation in muscles and acquired 3D time-series images of muscles in metamorphosis using laser scanning confocal microscopy. To quantify the phenotypic effects of gene perturbations, we designed the Fly Muscle Analysis tool (FMAj) which is based on the ImageJ and MySQL frameworks for image processing and data storage, respectively. The image analysis pipeline of FMAj contains three modules. The first module assists in adding annotations to time-lapse datasets, such as genotypes, experimental parameters and temporal reference points, which are used to compare different datasets. The second module performs segmentation and feature extraction of muscle cells and nuclei. Users can provide annotations to the detected objects, such as muscle identities and anatomical information. The third module performs comparative quantitative analysis of muscle phenotypes. We applied our tool to the phenotypic characterization of two atrophy related genes that were silenced by RNA interference. Reduction of *Drosophila Tor *(Target of Rapamycin) expression resulted in enhanced atrophy compared to control, while inhibition of the autophagy factor *Atg9 *caused suppression of atrophy and enlarged muscle fibers of abnormal morphology. FMAj enabled us to monitor the progression of atrophic and hypertrophic phenotypes of individual muscles throughout metamorphosis.

**Conclusions:**

We designed a new tool to visualize and quantify morphological changes of muscles in time-lapse images of *Drosophila *metamorphosis. Our *in vivo *imaging experiments revealed that evolutionarily conserved genes involved in *Tor *signalling and autophagy, perform similar functions in regulating muscle mass in mammals and *Drosophila*. Extending our approach to a genome-wide scale has the potential to identify new genes involved in muscle size regulation.

## Background

Muscle wasting occurs in ageing, immobility and disease. In order to discover pharmacological cures for human muscle wasting disorders like cachexia and sarcopenia the regulation of skeletal muscle mass has been studied extensively [1]. Muscle mass is regulated by balancing protein synthesis and degradation [2] which can either occur via ubiquitin mediated proteolysis or autophagy [3]. Protein synthesis and cell growth are promoted by a pathway consisting of the insulin-like growth factor *IGF1*, the kinase *Akt *and the mammalian target of rapamycin (*mTOR*). *Akt *represses the atrophy promoting transcription factor FoxO, while *mTOR *stimulates protein translation and inhibits autophagy [4]. In contrast, muscle atrophy is activated through the Myostatin and Smad3 signalling pathway, which activates ubiquitin dependent proteolysis via the FoxO [5]. Most of our knowledge about muscle size control and atrophy is derived from C2C12 myoblast cell culture and mouse transgenic models [6]. However, not many studies have used *in vivo *imaging, which depends on the ability to non-invasively observe muscle fibers in their natural environment combined with genetics as an experimental tool.

*Drosophila melanogaster*, henceforth referred to as *Drosophila*, displays a holometabolous life cycle. Metamorphosis transforms larval into adult body structures in approximately 4-5 days and involves cell death, remodelling and proliferation [7]. Head eversion (HE) occurs 12 hours after puparium formation (APF) and constitutes the prepupal to pupal transition (PPT) that gives rise to the three major body parts (head, thorax, abdomen) of adult flies. In this study, we focus on two types of larval abdominal muscles; dorsal external oblique muscles (DEOMs) which undergo histolysis prior to HE and the dorsal internal oblique muscles (DIOMs) which are remodelled into adult muscles [8,9]. The change in morphology and position of persistent DIOMs after HE is accompanied by an atrophy-like decrease of muscle fiber diameter and an increase of diameter prior to eclosion. Thanks to the transparency of *Drosophila *pupa and the availability of genetic tools like the UAS-GAL4 expression system [10], fluorescent reporters and reagents for RNA interference (RNAi) [11,12], it is possible to perform targeted gene perturbation and reporter gene expression. In a previous study, we introduced a custom pipeline to visualize and quantify 3D time-series images [9]. In a case study, we showed that a truncated GFP tagged version of nuclear EAST protein inhibited the histolysis of muscle fibers. Our current goal is to scale up this experimental approach to a larger number of genetic perturbations by taking advantage of publicly available transgenic lines. Although the previous segmentation tool could produce promising results using 3D images as inputs, it could not handle larger number of datasets.

To build a pipeline consisting of image processing, segmentation, relational database management and statistical analysis, and minimize the amount of manual data processing, we decided to develop a custom software tool. In recent years, many excellent non-commercial software packages for generic image analysis were developed, including ImageJ [13], Fiji [14], CellProfiler [15], BioImageXD [16], Icy [17] and Endrov [18]. Since these tools could not efficiently perform all required tasks, we designed the novel Fly Muscle Analysis in Java (FMAj) tool which is based on ImageJ. This tool processes 2D projection instead of 3D stacks for high throughput analysis of time-series images. The FMAj tool consists of three modules for image processing and annotation, segmentation and quantitative phenotypic analysis. An important feature of this tool is the incorporation of a relational database, MySQL, to store the segmented regions of interest (ROIs) along with extracted features. We demonstrated the image analysis capability of FMAj by phenotypic characterization of targeted gene silencing by RNAi of *Tor *and *Atg9*.

## Methods

### Microscopy

All *Drosophila *strains were kept at 25^o^C. *MHC-tau-GFP *[19] was used to label muscle cytoplasm and *UAS-Histone 2Av-mKO *[20] to label nuclei. *Mef2-GAL4 *was used as a muscle specific driver [12]. The females from reporter line were crossed with males from UAS-shRNA (small hairpin) transgenic lines obtained from the Transgenic RNAi Project (TRiP) collection [21]. The genotype of the reporter line was *MHC-tau-GFP/FM7-GFP*; *Mef2-GAL4, UAS-histone-mKO/TM6B Tb*. The progeny of *MHC-tau-GFP/+; Mef2-GAL4, UAS-histone-mKO/UAS-GeneX-RNAi *genotype, i.e. non-*Tubby *pupae expressing both live reporters, were used to examine the muscles. In this study, we used RNAi lines of the following genes: *Chromator *(Control, Bloomington Stock id: B-36084), *Atg9 *(B-34901) and *Tor *(B-35578).

Sample preparation and microscopy were performed as previously described [9]. Samples were collected at the white pupal stage, washed with water to remove the fly food from their surface and inspected under a fluorescent stereomicroscope (Olympus MVX10, Olympus, Tokyo, Japan) to select prepupae expressing both reporter genes. Up to 30 prepupae were placed on an uncoated 32 mm diameter glass bottom dish (MatTek, Ashland, Massachusetts), with the dorsal side directed towards the bottom of the dish. The prepupae were mounted in CyGEL (Biostatus Ltd, Leicester, UK) to restrict their movement during imaging. A wet tissue was kept around the specimens to maintain humidity levels during imaging.

We used the line scanning Zeiss LSM 5 Live microscope with a motorized stage to perform multi-location live imaging of *Drosophila *pupae. For each sample, we collected images at multiple focal planes and multiple time points. The Zen 2008 software was used to configure the settings of the confocal microscope. We acquired the images with the following settings; 10 × magnification (EC Plan-Neofluar 10×/0.30 M27), pin hole size of 16.6 μm and frame speed of 2 FPS. The images were collected at a frame size of 1024 × 1024 pixels; with optical slices ranging from 30 to 40. The physical size of pixel was 1.25 µm and the distance between focal planes 11.08 µm. Because single dish can accommodate multiple samples, the acquisition software was configured to collect images at multiple locations. We used a data logger device to record temperature and humidity during imaging. Image stacks were acquired at 30 minute intervals. The confocal images (8 bit) were stored in LSM format (Carl Zeiss) with one LSM file per time point and sample. The LSM files of each sample were concatenated and stored as ICS files using custom software [22]. Subsequently, 3D stacks of ICS files were converted to maximum intensity projections (MIP) and saved as multi-page TIFF files. Pages in multi-page TIFF files represented time points and served as the inputs of the FMAj tool.

### Image processing using FMAj

#### Image analysis workflow of the FMAj tool

**Figure 1:**
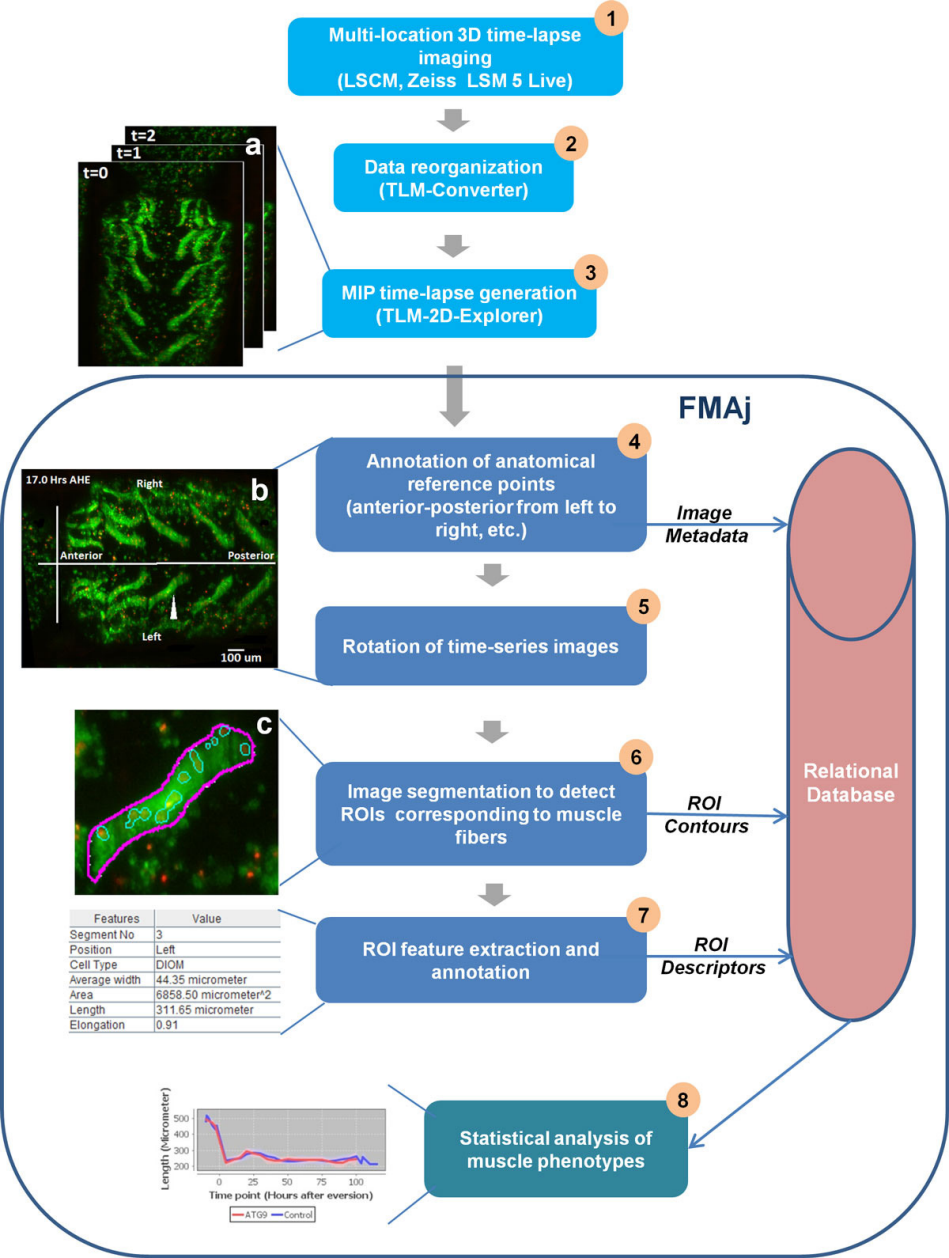
**Workflow of the image analysis pipeline**. Image processing upstream of FMAj consists of three steps. 3D time-lapse image data acquired by laser scanning confocal microscopy (a) are concatenated using the TLM-Converter custom software (step 2). Another custom software, called TLM-2D-Explorer (step 3), generates MIPs of 3D stacks, each of which represents a time point. The workflow in FMAj consists of 5 major steps. Note in the text, steps 4 and 5 are referred to as module 1, 6 and 7 as module 2 and 8 as module 3. (step 4) The user interactively annotates the anatomical reference points like anterior-posterior axis, left-right axis and the temporal reference points, such as the time of head eversion (b). The annotation details and metadata collected from the microscopic images are stored in the database. (step 5) To facilitate visual comparison of different time-lapse datasets, the anterior-posterior axis is rotated to be shown from left to right in the horizontal orientation. (step 6) Manual and semi-automated segmentation methods based on level-set help to define the contours of muscle fibers recorded in the green channel. In an optional step, nuclei recorded in the red channel can be segmented using an automated method. (c) Magnification of the segmented muscle indicated by an arrowhead in (b) and its nuclei. The ROIs corresponding to muscle fibers and their nuclei are stored in the database. (step 7) Shape and size features are calculated from the boundaries of the ROIs and stored into the database. ROIs are manually assigned muscle types for subsequent tracking and time-series analysis. (step 8) Tracks of ROIs along with their feature values are retrieved from the database to perform a statistical analysis of muscle phenotypes.

**Figure 2:**
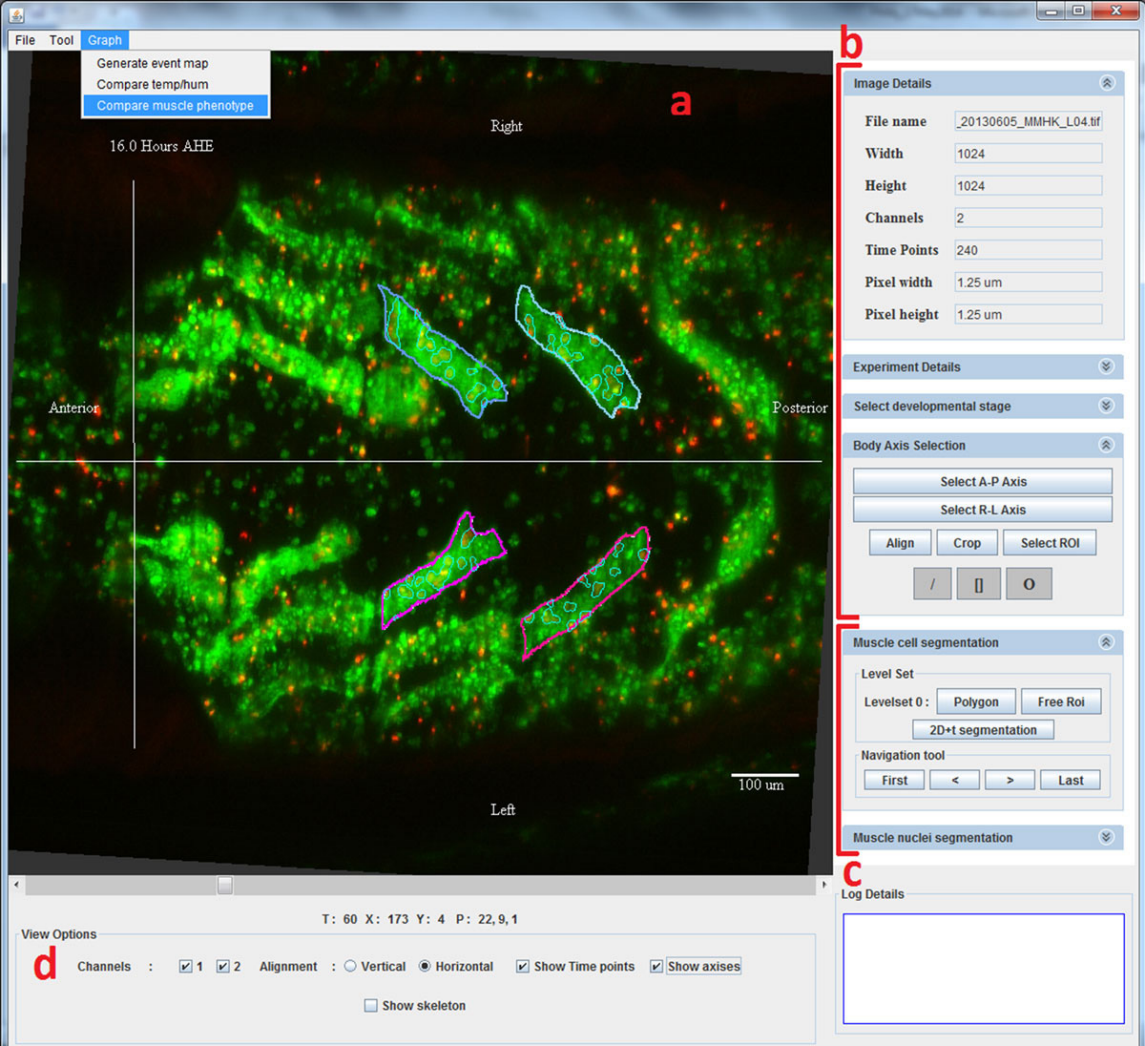
**Screenshot of the FMAj user interface**. (a) In the left panel, the user can browse through the time-lapse images. Various annotations can be projected onto the images, including anterior-to-posterior and left-to-right body axes, scale bar, time point and contours of segmented ROIs. (b) The top right panel shows image metadata and contains controls for manual annotations. (c) The bottom right panel contains the control elements for manual and semi-automatic segmentation. Annotations like muscle type can be added to selected ROIs. (d) The bottom panel controls view options like color channel display.

Figure 1 illustrates the image processing pipeline of FMAj. After starting the application, the user establishes a database connection and selects the root folder of the microscopic images. Once the database is online, the tool automatically downloads the experimental information, such as gene names, stock ids, muscle type, developmental stages and the imaging protocol from the MySQL database. Initially, metadata about image acquisition are extracted from raw image files, while biological details are entered into the database by an expert user. Input images of FMAj are time-series MIPs in RGB format containing two colour channels; with green representing the cytoplasm and red the nuclei of muscle fibers (Figure 2). The tool provides options to view both channels together or separately. The FMAj tool consists of three modules which perform three sequential tasks. The first module captures experimental metadata which are either derived from the images or via manual annotation by the user. An important user input is the definition of the onset of head eversion, which serves as the main reference point (time point = 0 hours) for comparing datasets. The second module performs segmentation of muscle cells and nuclei in a semi-automated fashion. Features of detected ROIs are either calculated (e.g. shape) or annotated by the expert user (e.g. cell nomenclature). The third module performs comparative phenotypic analysis, such as comparing the cell morphology between control and genetically perturbed cells.

### Module 1: Annotation of time-series images

When the user opens a multi-page TIFF image stack in FMAj, the tool retrieves image metadata like width, height, physical pixel size and bit depth. The user can view the image acquisition settings extracted from LSM metadata like magnification, laser wavelength and pinhole size. The user can also upload temperature and humidity readings collected using a temperature logger device from a spreadsheet to the database. The assay details like fluorophore, genotype (transgenes), stock id and sample location are manually entered by the user.

To facilitate the objective comparison of image sets of different samples, we established a robust spatial and temporal reference system. Spatial registration is achieved by interactively drawing a left-right symmetry axis along the midline and rotating the time-lapse image stack so that the anterior to posterior axis of the pupa is oriented horizontally from left to right. In addition, the user draws a second line that demarcates the boundary between thorax and abdomen. These reference lines assist in the visual comparison of different samples. The onset of head eversion occurs approximately 12 hours after puparium formation and was used for the temporal alignment of different samples, thus enabling the comparison of equivalent frames in different time-lapse datasets [9].

### Module 2: Segmentation of muscle cells

The goal of image segmentation is to quantify the morphological changes of persistent abdominal muscles throughout metamorphosis. In the early pupal stages right after head eversion, muscles are difficult, if not impossible, to segment using automated methods due to large amounts of debris created by the histolysis of obsolete muscles. Therefore, we decided against an automatic approach for muscle detection. Instead, we applied a semi-automated active level set method [23] for segmentation of muscle cells. In the first step, the user draws one or more polygons around muscles of interest in one or more frames. In the second step, the polygons serve as initial level sets for the subsequent curve evolution, which is performed by an ImageJ level set plugin [24]. The contour evolution is controlled by an edge-based constraint that applies grey value and curvature penalties to prevent the leakage of contours into low contrast regions. For muscle cell segmentation, contour evolution is restricted to the inside of manually drawn polygons. If the output of the level-set method is not satisfactory, the user can delete the ROI and manually redraw its boundary using ImageJ editing functions.

For each segmented muscle in a particular frame, the user assigns a unique description based on 4 criteria; (1) the cell type (e.g. DEOM or DIOM), (2) the body part (e.g. thorax or abdomen), (3) the segment (e.g. abdominal segment 3) and (4) the lateral position (left or right hemi-segment). To improve productivity, lateral positions can be inferred from the midline, while tracking of overlapping ROIs provides the options to automatically propagate annotations to other subsequent frames. Correspondences between cells in adjacent time points are established using a nearest neighbour search on the basis of minimum distance between centroids. To avoid incorrect tracking, a maximum distance *D *between centroids of adjacent frames is used as a constraint. We estimate *D *based on the maximum movement of muscle cells observed in 30 minute intervals. Highlighting different cell types with different contour colours helps to identify annotation errors that can be manually corrected by the user.

### Nuclear segmentation

Muscle cells are multinucleated. Since the images were recorded at a relatively low resolution using a 10× objective, we often had difficulties identifying individual nuclei. Hence, instead of identifying the centroids of all nuclei, we modified our goal to detect the regions (or clusters of nuclei) within muscle fiber. Nuclear regions are detected by applying Otsu adaptive thresholding to ROIs of muscles [25]. The boundaries of nuclei can be viewed as contours within muscle ROIs in FMAj (Figures 1c, 2a).

### Feature extraction from regions of interest

**Figure 3:**
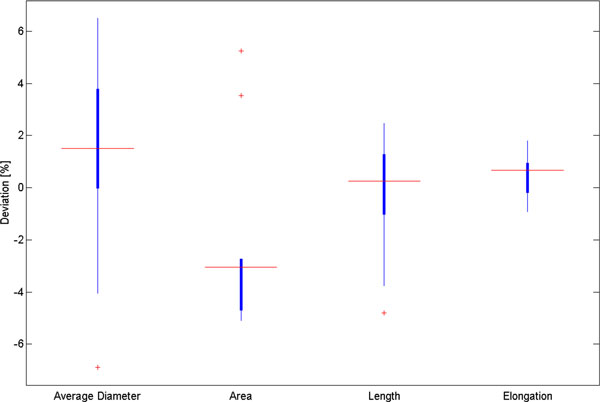
**Validation of segmentation and feature measurement in FMAj**. The boxplots show the percentage deviations of feature values between FMAj and Fiji following segmentation and feature calculation of 10 muscles using the respective tools.

The difference in muscle shape and size between mutants can be quantified by comparing various morphological features extracted from muscle cell contours. The collection of features available in FMAj are average diameter, area, length, circularity, elongation, orientation, extent, roundness, aspect ratio and solidity [26]. Average diameter is calculated by measuring the width of muscle cell at multiple points along its skeleton [27], which is equivalent to the medial axis of the muscle cell. Average diameter, area and length relate to size, while elongation, extent, roundness and solidity describe the shape of muscle cells. The accuracy of segmentation and feature measurements in FMAj was validated using the Fiji image analysis package. After manually segmenting 10 ROIs using the segmentation editor plugin, we determined the average diameter by manually measuring the width at 5 locations perpendicular to the medial axis. Length and area were calculated using Fiji, elongation using the regionprops function in MATLAB. The median deviations of 4 features between FMAj and external tools ranged from -4% to +2% (Figure 3).

### Module 3: Quantitative phenotypic analysis

#### Data browsing

FMAj allows the user to view images, annotations and processed data. To detect interesting phenotypes, spatially and temporally aligned datasets can be viewed side by side. Annotations such as boundaries can be projected on top of images.

### Statistical analysis

To compare muscle phenotypes of different RNAi genotypes, we segmented muscles in abdominal segments 3-5 of 10 pupae per genotype at 5 hour intervals. With one muscle per hemi-segment, our statistical analysis comprised 10-20 muscles per genotype and time point. To compare the morphology of different genotypes at equivalent times in development, we performed the non-parametric Mann-Whitney U test in FMAj with help of the Java Statistical Classes (JSC) library [28]. Significance values (p) were determined using a non-parametric test because of low number of samples per population (10-20) and unknown distributions. The Mann-Whitney U test statistics can be viewed in graphical form using the phenotype comparison tool. For ease of visualization, p-values are plotted in a -1*log_10_(p-value) scale.

### Graphical visualization of time-series data

FMAj uses the JFreeChart [29] library to generate charts, such as line plots, to visualize and compare the dynamics of cellular features. The user interface allows users to select the genotypes, muscle types and shape features. When comparing different populations, mean or median values along with their error margins (standard deviations, 25-75% percentiles) can be plotted. Significance values can be displayed underneath the charts of cellular features. Environmental parameters like temperature and humidity can also be plotted.

### Database management

**Figure 4:**
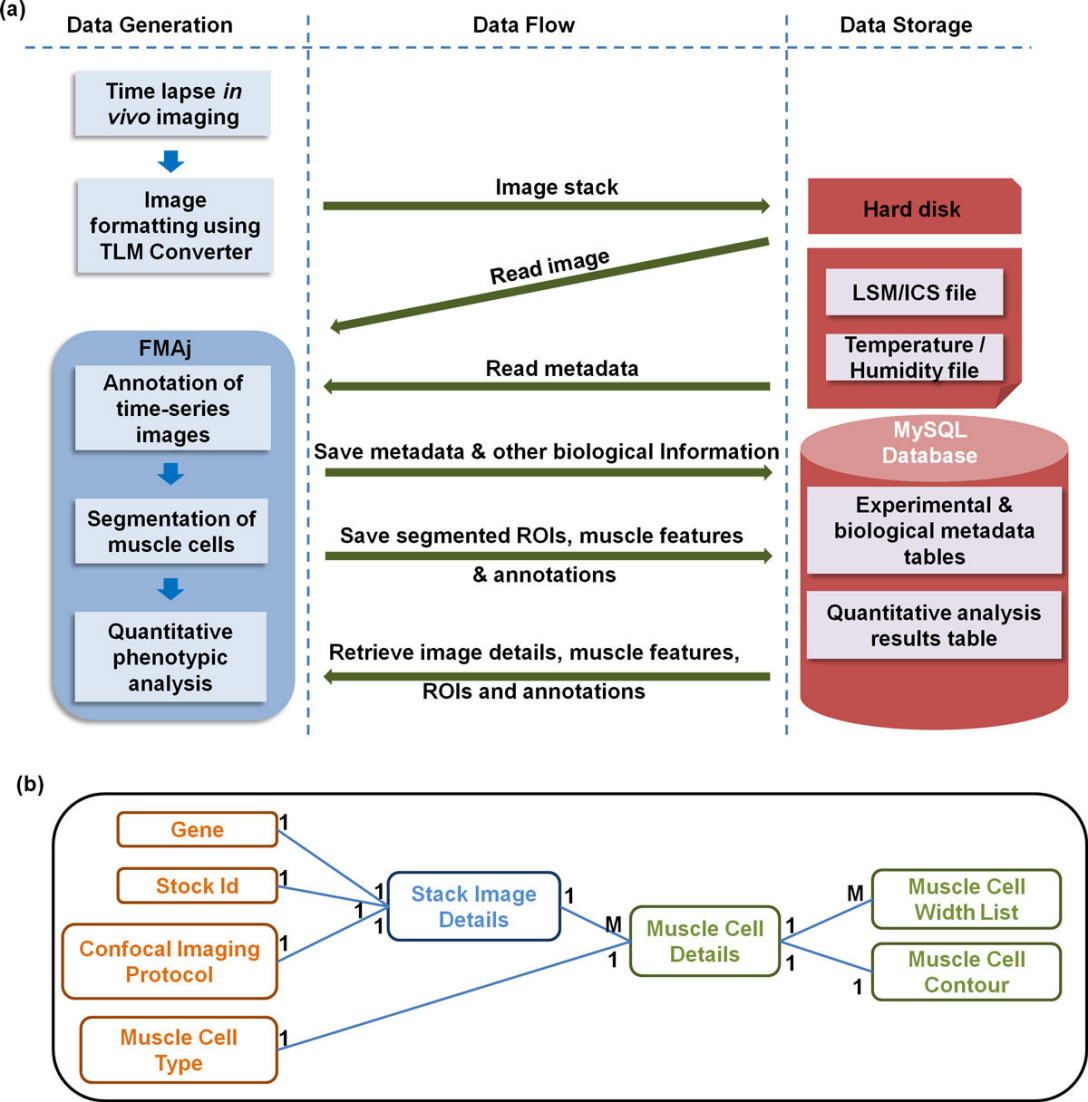
**Schematic diagram of the dataflow between FMAj and the relational database**. (a) FMAj generates two types of image descriptors that are stored in the database. First, metadata of the time-series images contain biological parameters of the live samples (e.g. genotype and fluorescent marker) and details about the imaging experiment (e.g. objective, laser excitation wavelength, pixel size). Second, descriptors of segmented muscle ROIs contain polygons, shape features, annotations provided by the user (e.g. cell type) and tracks, i.e. assignments between ROIs in subsequent frames. In the data flow diagram, forward arrows indicate storage of data while backward arrows indicate retrieval of data. Raw and processed images are stored on the local hard disk. Their file locations and metadata are stored inside the MySQL database. (b) Simplified version of the entity relationship diagram of our database. Each block represents a table and each line indicates a relationship between tables. The notations at the end of lines define the type of relationship, such as one (1) to one (1) or one to many (M). For example, the muscle cell details table has a *One to One *relationship with muscle cell type. Table names in orange store experimental and biological metadata, while those in green store results from image segmentation like the annotation or calculated features of muscle ROIs.

To improve data handling efficiency, we created a relational database using MySQL and connected it to FMAj. Figure 4a shows the general dataflow between FMAj and the database. MySQL is an open source database which ensures data integrity and is very efficient in querying large amount of data [30]. The dependencies and relationships between the entities of our experimental data were modelled in the relational database tables (Figure 4b).

The database is organized into two groups of tables; one for experimental and biological metadata and another one for segmentation outputs. Data collected in module 1 is stored in experimental and biological metadata tables. It contains two types of data. First, experimental parameters like genotypes and imaging protocols are entered directly into the database by the user. Second, image acquisition parameters like laser wavelength, magnification and image properties like stack name, image size, bit size, physical size of pixel are extracted from LSM image files recorded by the microscopy system. Results produced by the segmentation module 2 are stored in ROI tables. These tables store ROIs corresponding to muscle cells and nuclei as blobs. They also store the features extracted from muscle cell boundaries. The image stacks are not stored in the database because of their large sizes. Instead, their file names and locations on the hard disk relative to a user-defined root folder are stored in the MySQL table *StackImageInfoMaster*. In order to make sure there is no redundancy of data, each stack can be identified by its name and a unique set of three parameters: stock id, date of acquisition and location of sample in the glass bottom dish. Module 3 of FMAj retrieves information from the database for image browsing or graphical comparison of different samples based on specific criteria of query.

## Results

**Figure 5:**
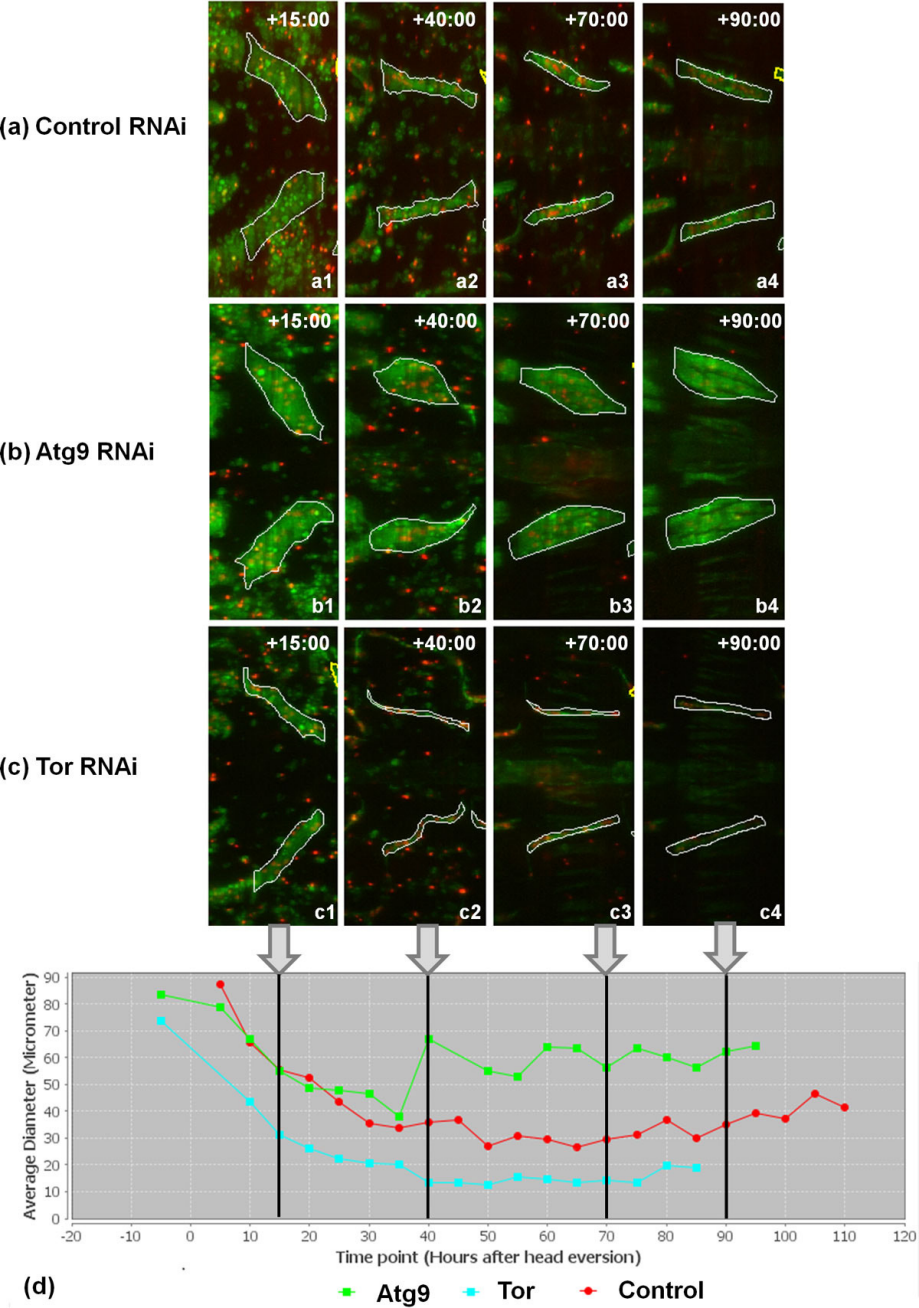
**Genetic perturbations of autophagy and the *Tor *pathway affect developmental atrophy of persistent muscles**. Short hairpin RNAs (*UAS-shRNA*) and the nuclear *UAS-histone-mKO *(red) reporter were co-expressed in muscles using the *Mef2-Gal4 *driver. Another fluorescent reporter *MHC-tau-GFP *(green) was used to label muscle cell bodies. (a) In a control pupa, persistent muscles in the 3^rd^ abdominal segments undergo atrophy upon head eversion which is defined as time point zero hours. (b) Silencing of *Atg9 *by RNAi inhibits atrophy, resulting in enlarged muscle fibers compared to control. (c) Silencing of *Tor *enhances atrophy, leading to thinner fibers. (d) The effects of gene perturbations on muscle fiber diameter can be compared in time-series plots.

**Figure 6:**
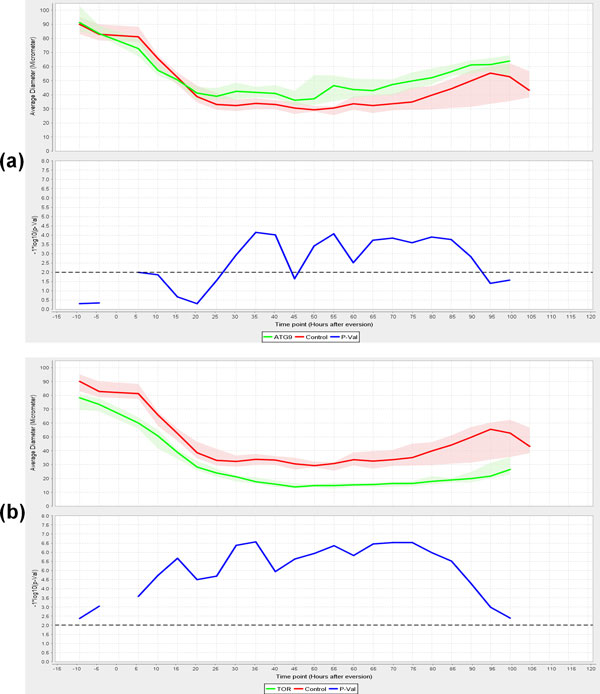
**Graphical comparisons of median diameter between different genotypes and their statistical significance**. The line charts (top panels in a and b) compare median muscle diameters of populations of muscles expressing different RNAi constructs. The data were collected over a period of 120 hours from 10 pupae per genotype. For each population, statistics were derived from 10 muscle cells. The red and green lines show the median diameter, while the adjacent shaded regions indicate 25^th^ and 75^th^ percentiles. The horizontal dotted lines in the significance graphs (bottom panels) represent the p-value 0.01. Due to the negative log scale, measurements of significant difference are shown above the line. (a) Silencing of *Atg9 *caused decreased muscle atrophy compared to controls from 30 hours AHE onwards. Prior to 30 hours AHE, no significant difference were observed between the two genotypes. (b) Silencing of *Tor *lead to an enhancement of developmental atrophy. The graph shows that the thinning of muscle fibers proceeds for a longer period (45 as opposed to 30 hours AHE) than in control.

**Figure 7:**
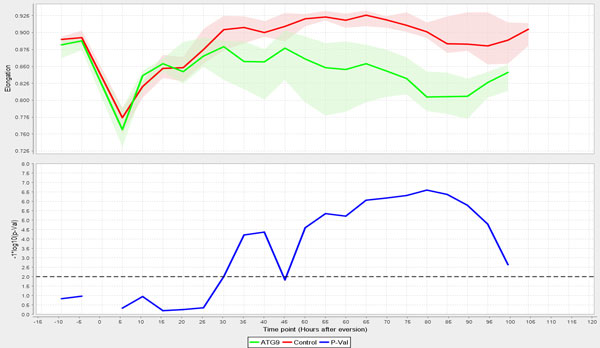
**Shape parameters derived from segmented muscles can help quantify differences in atrophy patterns**. The top chart compares the changes in the shape feature elongation between *Atg9 *and control RNAi expressing muscles. The ROIs used for the analysis were the same as in Figure 6a. In the first 25 hours AHE, both genotypes showed similar increases of elongation coinciding with muscle atrophy. In the next 70 hours, median elongation was significantly smaller in *Atg9 *compared to control muscles in almost all measurements, suggesting an inhibition of atrophy.

Autophagy, which is negatively regulated by *Tor *signalling, sequesters cytoplasmic proteins and organelles for lysosomal degradation [31]. In *Drosophila *metamorphosis, autophagy is believed to control the cell death of larval tissues like salivary gland, midgut and fat bodies [32]. In order to test FMAj in quantifying developmental changes in muscle morphology and explore the genetic control of muscle remodelling, we selected three UAS-shRNA (small hairpin RNA) RNA interference constructs crossed to reporter genes for *in vivo *time-lapse microscopy and image analysis. The constructs corresponding to *Tor*, *Atg9 *and *Chromator *(*Chro*) were chosen based on 3 different phenotypes observed in late pupae by stereomicroscopy. *Tor *RNAi produced smaller, while *Atg9 *resulted in enlarged muscles (Figure 5). *Chro *served as control since the muscles were indistinguishable from unperturbed muscles. We acquired 10 time-lapse datasets over 5 days per genotype, each of which contained 240 time points recorded at 30 minute intervals from the prepupal until the pharate adult stage. To monitor developmental changes of DIOMs, we segmented muscle fibers at 5 hours intervals (Figure 5a-c) and determined their areas (not shown) and average diameters (Figure 5d). In mammalian models, cross sectional area or diameter is the main feature to quantify muscle atrophy. In the control animal (Figure 5a), diameter decreased approximately 3-fold from 90 to 30 µm in the first 50 hours AHE. Consistent with its role in promoting growth, *Tor *silencing (Figure 5c) resulted in smaller muscles throughout metamorphosis, suggesting enhanced atrophy. In contrast, inhibition of the autophagy factor *Atg9 *did not lead to altered diameter compared to control until 30 hours AHE. An enlargement of the muscle became only apparent in the later stages. Although removal of many larval tissues is believed to be mediated by autophagic cell death, the loss of *Atg9 *did not cause delay of DEOM histolysis, as was observed in the case of EAST overexpression [9]. The different temporal profiles in muscle remodelling were also seen when comparing populations of muscles from 10 animals per genotype (Figure 6). For each time point, we determined the median diameter of 10-20 muscles. To visualize the range of features, FMAj can display the 25% and 75% percentiles around the median value. Statistical differences between control and the two knockdowns were calculated using the Mann-Whitney U test and plotted beneath the line charts showing muscle diameters. In the case of *Atg9 *RNAi (Figure 6a) versus control, we observed two phases of remodelling. In the early phase up to 30 hours AHE, when muscle diameters appear similar, the p-values remained above a threshold of 0.01. At 30 hours AHE and later the median diameters diverged, with *Atg9 *silencing resulting in a suppression of atrophy compared to controls. This divergence was reflected by lower p-values. The time-series plot comparing population medians of controls with *Tor *RNAi (Figure 6b) revealed a prolonged atrophy phase from 0 to 45 hours AHE in *Tor *mutants compared to 0-30 hours AHE in controls. As the diameters were discernibly different throughout metamorphosis the p-values remained below the 0.01 threshold. Besides an increase in cell size, *Atg9 *RNAi also caused a change in shape of muscle cells. From the mid-pupa stage onwards (30 h AHE), we observed that the muscle cells were thicker in central than terminal regions, (Figure 5b) indicating that autophagy is not only attenuated but also unevenly distributed along the longitudinal axis of fibers. In comparison, control muscles were thinner with even diameter (Figure 5a; 70 and 90 hours AHE). To quantify the attenuation of muscle thinning, we calculated elongation, which is defined as the ratio between the difference in length of major and minor axis and length of major axis. In early pupa (0-25 hours AHE) of control and *Atg9 *RNAi genotypes, elongation showed a steady increase as muscles underwent thinning along their longitudinal axis (Figure 7, top panel). From midpupal stage onwards, the curves of median elongation values continued increasing in controls, while decreasing in *Atg9*, indicating a suppression of atrophy. This divergence was reflected by a decrease in significance values (Figure 7, bottom panel) determined by the Mann-Whitney U test.

## Discussion

The ability of fluorescent markers to visualize a specific biological process without affecting the global physiology of cell has made it an ideal tool for studying gene functions. We have used *in vivo *time-lapse imaging to study the effects of gene perturbation on developmental muscle atrophy in *Drosophila *metamorphosis. We developed the FMAj software tool for the quantitative characterization of muscle phenotypes in time-series images. To effectively perform comparative phenotypic profiling of muscle development, we integrated image processing, segmentation, structured storage by MySQL and statistical analysis. The integration of multiple tasks enhances productivity as the alternative export of ROI data and the manual processing in a spreadsheet program would be much more time-consuming. A major motivation of this study was to visualize and quantify alterations in muscle fiber size and shape in response to genetic perturbations. Relative to controls, silencing of *Tor *resulted in enhanced developmental atrophy and smaller muscles, while silencing of *Atg9 *led to an inhibition of atrophy and enlarged muscles. These results on gene function are new in the context of *Drosophila *metamorphosis, yet in the wider context of cell size regulation, they are not unexpected since signalling pathways involving *IGF1*, *Akt *and *mTOR *are well-known to promote protein synthesis and the growth of many cell types, including muscles in mammals [33] and *Drosophila *[34]. We show that our system can help to fill some knowledge gaps and propose new hypotheses that can be further tested experimentally. Although it is well-established that autophagy plays an important part in protein degradation during muscle atrophy [4,35], there is little, if any, evidence that *Atg9*, a transmembrane protein involved in autophagosome formation [36,37] participates in the control of muscle size. Furthermore, the case of *Atg9 *demonstrates how time-series analysis can uncover a transient phenotype that may have been missed by traditional end-point assays. Besides enlarged muscle fibers in late pupae, we could show that muscle size only starts to deviate from controls 20 hours AHE, indicating that *Atg9 *function may not be required for atrophy in this early phase of metamorphosis. The temporal profile of *Tor *RNAi, besides the obvious muscle size reduction, showed that atrophy progressed for a longer period in persistent muscle, suggesting that *Tor *may act to inhibit atrophy around 30 hours AHE. Expanding our approach of combining *in vivo *imaging with quantitative analysis to a genome-wide scale has the potential to uncover new players of *Drosophila *muscle remodelling, some of which may also turn out to be novel factors in mammalian muscle size control. In summary, our model can fill knowledge gaps and propose new hypotheses in the arena of muscle wasting research.

Since FMAj takes advantage of the ImageJ library, it will be easier to enhance its functionalities by incorporating additional image processing and computer vision algorithms. A drawback of the current FMAj version is that ROI detection is performed in a manual fashion. To enhance throughput, we plan to propagate initial contours to subsequent time points. Moreover, we plan to use a multivariate shape feature analysis to study the developmental changes of muscle morphology.

## List of abbreviations used

FMAj: fly muscle analysis in java; DEOM: dorsal external oblique muscle; DIOM: dorsal internal oblique muscle; GFP: Green Fluorescent Protein; MIP: Maximum intensity projection; mKO: monomeric Kusabira Orange; MHC: Myosin heavy chain; TOR: Target of rapamycin; AHE: After Head Eversion; APF: After puparium formation; TLM: time-lapse microscopy; ROI: region of interest; RNAi: RNA interference

## Competing interests

The authors declare that they have no competing interests.

## Authors' contributions

K was involved in designing and implementing FMAj, image processing, laboratory experiments and drafting the manuscript. WC was involved in laboratory experiments and microscopy. LF provided academic advice on K's research work in image processing and algorithm design. MW conceived and designed the study, was involved in designing and implementing FMAj, image processing, laboratory experiments, microscopy and drafting the manuscript.
